# 1622. Increase in Congenital Toxoplasmosis and Acute Toxoplasmosis Among Pregnant Women in the US during the COVID-19 pandemic

**DOI:** 10.1093/ofid/ofad500.1457

**Published:** 2023-11-27

**Authors:** Despina Contopoulos-Ioannidis, Valerie Bonetti, Jose G Montoya

**Affiliations:** Stanford University, School of Medicine, Stanford, CA; Dr. Jack S. Remington Laboratory for Specialty Diagnostics, Palo Alto Medical Foundation, Sutter Health, Palo Alto, CA, USA, Palo Alto, California; Dr. Jack S. Remington Laboratory for Specialty Diagnostics, Palo Alto Medical Foundation, Sutter Health, Palo Alto, CA, USA, Palo Alto, California

## Abstract

**Background:**

During the COVID19 pandemic a rise in diverse opportunistic infections has been described

**Methods:**

In this retrospective cohort study, we analyzed the trajectory of new congenital toxoplasmosis (CT) cases and cases of acute toxoplasmosis among pregnant women (AT-P) during the COVID19-pandemic (2020-2022) compared to a pre-pandemic period (2000-2019). We perused data from the Remington’s Laboratory, the National Reference Laboratory for Toxoplasmosis in the US. We calculated the yearly number of cases of CT and AT-P; the incidence rate (IR) of CT and AT-P (IR=number of cases/total number of children < 12 months or pregnant women respectively tested by the *Toxoplasma* IgG-Dye test). We used ARIMA time-series models to compare the trajectory slopes between the two periods.

**Results:**

In the pre-pandemic period there were 196 CT cases among 8194 tested children< 12 months and 814 AT-P cases among 33,345 pregnant women. The respective numbers during the pandemic were 43 CT among 1833 tested children < 12 months and 65 AT-P among 1983 pregnant women. In 2022 we had the largest number of CT cases (21 CT in 2022 vs 6 in 2019) and similarly a high number of AT-P cases (22 AT-P in 2022 vs 16 in 2019)(Fig 1). This increase occurred, despite a drop in the total number of samples tested in the Lab ≥2020, due to programmatic changes in one major Lab client (Fig 2). The yearly IR of CT increased during the pandemic, from 1.05 cases/100 tested (6/574) in 2019 to 3.71 cases/ 100 tested (21/566) in 2022. The yearly IR of AT-P also increased during the pandemic, from 1.35 cases/ 100 tested (16/1183) in 2019 to 3.79 cases/100 tested (22/580) in 2022 (Fig 3). There was a positive trajectory-slope trend in CT and AT-P during the pandemic. This occurred after a preceding negative trajectory-slope trend in AT-P in the pre-pandemic period.

**Figure 1:**
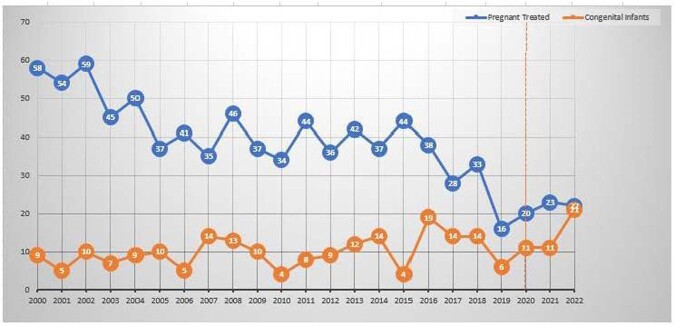
Number of Congenital Toxoplasmosis and Acute Toxoplasmosis during Pregnancy cases: 2000-2022

**Figure 2:**
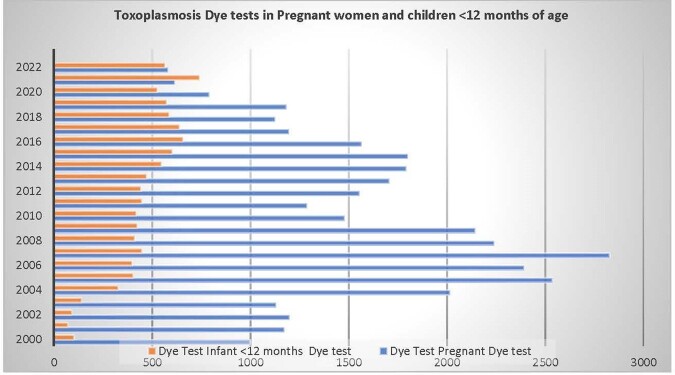
Toxoplasmosis IgG Dye tests in Pregnant Women and in Children <12 months of age: 2000-2022

**Figure 3:**
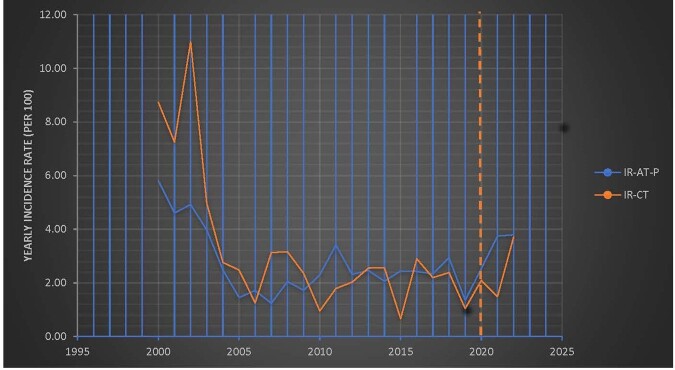
Incidence Rates of Congenital Toxoplasmosis (IR-CT)and Acute Toxoplasmosis During Pregnancy (IR-AT-P): 2000-2022

**Conclusion:**

During the pandemic, there was a rising trend in CT and AT-P cases diagnosed in the National Reference Lab for toxoplasmosis in the US. In 2022 there was a peak in the CT cases. The etiology remains unclear. A possible increase- during the lockdowns-in the contact of pregnant women with cats and/or a possible limited prenatal-preventive-guidance during the pandemic due to possible disruption in prenatal care services, could have been contributing factors. These trends need further monitoring.

**Disclosures:**

**All Authors**: No reported disclosures

